# Diagnosis of *Chlamydia psittaci* pneumonia using targeted next-generation sequencing: case report and clinical characteristics statistics

**DOI:** 10.3389/fmed.2025.1662623

**Published:** 2025-10-03

**Authors:** Miao Yu, Wenbin Wang, Huajuan Zhou, Amit Kumar, Xiaoli Zhao, Fengfei Qian, Xiaohua Zhong, Chuanhua Nie, Roy Sukanya

**Affiliations:** ^1^Pulmonary and Critical Care Medicine, Hangzhou Linping District Hospital of Integrated Traditional Chinese and Western Medicine, Hangzhou, China; ^2^Wenzhou Medical University Renji College, Wenzhou, China; ^3^School of International Studies, Wenzhou Medical University, Wenzhou, China

**Keywords:** *Chlamydia psittaci*, pneumonia, targeted next-generation sequencing, case report, clinical features

## Abstract

*Chlamydia psittaci* (Cps) is a Gram-negative intracellular bacterium with birds as its main host. Humans are mainly infected through contact with birds and poultry. The main route is through inhalation of aerosols contaminated by animal excrement through the respiratory tract. The main manifestation after infection is pneumonia, and in severe cases, it can lead to multiple organ failure. Targeted Next-generation Sequencing (tNGS) can detect rare pathogens that are difficult to detect with traditional detection methods, which has continuously improved the detection rate of Cps. This article reports 12 cases of psittacosis pneumonia diagnosed by tNGS of bronchoalveolar lavage fluid (BALF) obtained by bronchoscopy or sputum. The cases are improved after regular treatment, and the clinical characteristics are analyzed at the same time.

## 1 Introduction

Psittacosis is a zoonosis caused by *Chlamydia psittaci* (Cps), a Gram-negative intracellular bacterium. Human infection is generally caused by ingestion of aerosols contaminated by the excreta of infected birds and poultry. The severity of human psittacosis infection can range from mild flu-like symptoms to life-threatening severe pneumonia and multiple organ failure (1). Cps is a rare pathogen of community-acquired pneumonia, accounting for only about 1% (2). Due to the non-specific clinical manifestations and limitations of detection methods, it is easy to miss or misdiagnose in the early stage. If the disease is not well controlled, pneumonia can progress rapidly and be complicated to multiple organ failure, which can be life-threatening in severe cases (3). This article reports 12 cases, including 10 mild to moderate pneumonia and 2 severe pneumonia. Targeted Next-generation Sequencing (tNGS) was used to diagnose psittacosis pneumonia, and all patients recovered and were discharged after regular treatment. At the same time, clinical characteristics Statistics and relevant literature review are carried out to provide reference, diagnosis and treatment ideas for clinical physicians, so as to enable early detection, early diagnosis and early standardized treatment of the disease.

## 2 Method

The Multiple PCR-based targeted NGS generally includes three main stages: sample and lab processing, sequencing, and data analysis. In the first stage, patient samples are collected and nucleic acids are extracted through lysis, purification, quantification, and quality checking. High-quality DNA or RNA is then used to prepare sequencing libraries, where the target regions of interest are enriched and unique barcodes are added to each sample. After library quality control (QC), sequencing is carried out on a next-generation sequencing platform, producing raw reads. For the data analysis part, the raw sequencing reads are first separated by sample, trimmed for low-quality bases, and aligned to the reference genome. Duplicates are marked or UMIs collapsed when needed. Variants are then called, filtered to remove technical noise, and annotated with reference databases. QC metrics, including coverage depth, on-target rate, and uniformity, are evaluated to ensure reliable results. Finally, clinically relevant variants are summarized in a report and interpreted according to established guidelines, supporting accurate molecular diagnosis. Bronchoalveolar lavage fluid (BALF) tNGS testing for all 12 patient was performed by Hangzhou kingmed for clinical laboratory. Methodology: Multiple PCR-based targeted NGS is performed by making a panel of the specific sequences of prescreened pathogens, amplifying the target genes, obtaining information about the enriched nucleic acids through a high-throughput sequencing platform, and then analyzing the results by bioinformatics to identify pathogens. The specific data can be found in Table 1.

**Table 1:** Results of tNGS testing for *Chlamydia psittaci* in nine patients.

Patient number	Classification	Genus	Species	Normalized read count	Estimated pathogen load (copies/mL)
2	Mycoplasma/Chlamydia	Chlamydia	*Chlamydia psittaci*	7,716	5.5 × 10^5^
5	Mycoplasma/Chlamydia	Chlamydia	*Chlamydia psittaci*	11,637	>1.0 × 10^6^
6	Mycoplasma/Chlamydia	Chlamydia	*Chlamydia psittaci*	256	4.3 × 10^3^
7	Mycoplasma/Chlamydia	Chlamydia	*Chlamydia psittaci*	472	<1.0 × 10^3^
8	Mycoplasma/Chlamydia	Chlamydia	*Chlamydia psittaci*	43,922	>1.0 × 10^6^
9	Mycoplasma/Chlamydia	Chlamydia	*Chlamydia psittaci*	754	7.4 × 10^3^
10	Mycoplasma/Chlamydia	Chlamydia	*Chlamydia psittaci*	22,989	>1.0 × 10^6^
11	Mycoplasma/Chlamydia	Chlamydia	*Chlamydia psittaci*	1,672	1.1 × 10^4^
12	Mycoplasma/Chlamydia	Chlamydia	*Chlamydia psittaci*	10,376	>1.0 × 10^6^

## 3 Case report

### 3.1 Patient-1

A 47-year-old female patient was admitted to the hospital for fever accompanied by fatigue and muscle ache for 5 days. She had fever every day with the highest temperature of 39.9 °C, and gradually developed headache and shortness of breath after activities. Self-administered cephalosporin antibiotics were ineffective, so she came to our hospital for chest CT examination, which showed inflammation of the left lower lobe of the lung and a small amount of pleural effusion. Laboratory findings are summarized in Table 2. The fever subsided after 3 days of treatment with moxifloxacin (0.4 g, I.V., qd). The patient provided a medical history at admission and recently raised parrots. Since the patient refused bronchoscopy, sputum tNGS was sent for examination, and the results showed Cps. Considering that the symptoms had improved, the original treatment was continued for 10 days and then changed to minocycline (100 mg, P.O., q12h). The patient’s chest CT examination after 5 months showed that pneumonia has reduced.

**Table 2:** Laboratory test results of 12 patients with psittacosis pneumonia.

Patient number	WBC(x10^9^/L)	L(x10^9^/L)	CRP (mg/L)	PCT (ug/L)	LDH (U/L)	CK (U/L)	ALB (g/L)	Na (mmol/L)	ALT (U/L)	AST (U/L)
Patient-1	7.2	1.08	102.6	0.45	305	140	35	133	80	34
Patient-2	14.7	0.6	256.6	7.12	371	3,400	36	124	31	79
Patient-3	8.7	0.7	171.6	0.15	301	262	34	136	27	39
Patient-4	6.7	0.7	21.2	0.21	191	79	44	134	11	19
Patient-5	13.2	0.6	168	0.53	529	2,225	33	126	97	77
Patient-6	5.5	0.9	118.2	0.13	274	647	32	126	59	50
Patient-7	9.4	1.6	32	0.03	207	97	36	136	19	26
Patient-8	4.9	0.6	226.8	0.49	456	2011	30	133	28	53
Patient-9	9.4	0.7	96.6	0.21	206	37	33	132	63	52
Patient-10	7.3	0.7	261.3	0.67	329	591	34	131	27	44
Patient-11	4.3	0.5	136.2	0.9	165	53	34	134	22	30
Patient-12	7.8	0.6	76.2	0.33	446	1,550	30	130	80	52

WBC is white blood cell; L is lymphocyte; CRP is C-reactive protein; PCT is procalcitonin; LDH is lactate dehydrogenase; CK is creatine kinase; ALB is albumin; Na is blood sodium; ALT is alanine aminotransferase; AST is aspartate aminotransferase.

### 3.2 Patient-2

A 73-year-old male was admitted to the hospital for fever and cough for 3 days. The highest temperature was 40.1 °C, accompanied by fatigue, muscle ache, and loss of appetite. When he came to our hospital, a chest CT scan showed bilateral pneumonia and local atelectasis of the left upper lobe. Laboratory findings are summarized in Table 2. He still had recurrent high fever after 2 days of treatment with cefoperazone sulbactam (3 g, I.V., q12h). A bronchoscopy was performed to obtain bronchoalveolar lavage fluid (BALF) for tNGS examination, which showed Cps. After questioning the medical history, it was found that he had recently raised parrots. Therefore, after combined treatment with azithromycin (0.5 g, I.V., qd), the fever subsided and the symptoms gradually improved. After discharge, azithromycin (0.5 g, P.O., qd) was changed. A chest CT scan was repeated 1 month later, indicating that pneumonia has significantly reduced as compared to before.

### 3.3 Patient-3

A 66-year-old woman came to our hospital for fever and cough for 3 days. She had fever every day with the highest temperature of 39.8 °C, accompanied by muscle ache, headache, and vomiting. Self-administered cephalosporin antibiotics were ineffective, so she came to our hospital for chest CT, which showed infectious lesions in both lungs. Laboratory findings are summarized in Table 2. She still had recurrent high fever after 3 days of treatment with cefoperazone sulbactam (3 g, I.V., q12h). She underwent bronchoscopy to obtain BALF for tNGS, which showed Cps. After questioning her medical history, it was found that she had recently raised parrots, so she was treated with azithromycin (0.5 g, I.V., qd) and the fever subsided and the symptoms gradually improved. After discharge, she was changed to azithromycin (0.5 g, P.O., qd). A chest CT scan was repeated 1 month later, which showed that pneumonia has significantly reduced as compared to before.

### 3.4 Patient-4

A 55-year-old male was admitted to the hospital for fever for 4 days. He had fever every day with the highest temperature of 39.2 °C, fatigue and loss of appetite. Self-administration of cephalosporin antibiotics was ineffective, so he came to our hospital for chest CT examination, which showed inflammation of the left lower lobe of the lung Laboratory findings are summarized in Table 2. The fever subsided after 2 days of treatment with moxifloxacin (0.4 g, I.V., qd). After 1 week of treatment, the patient’s symptoms improved significantly and he was discharged from the hospital.

Subsequent epidemiological investigations showed that the above 4 cases were family cluster cases, with 2 parrots raised and the disease occurred within 1 month after the death of the parrots. Cases 1 and 4 were a couple, and cases 2 and 3 were a couple and the parents of case 1. The parrots had been raised in two families before they died. So, we contacted case 4 and suggested to complete tNGS, but the patient refused. Considering the history of contact with parrots and the other three family members all suffered from Cps pneumonia, combined with his own clinical manifestations and laboratory tests, the diagnosis was also considered Cps pneumonia, so the patient was recommended to be treated with minocycline (100 mg, P.O., q12h). After that, we contacted the patient to review the chest CT, but was refused.

### 3.5 Patient-5

A 77-year-old male patient presented with fever and cough for 3 days, accompanied by fatigue, muscle aches, hematuria, and diarrhea. He was then investigated with chest CT scan, which showed inflammation of the right upper lobe and bilateral pleural effusion. Laboratory findings are summarized in Table 2. The patient was still experiencing high fever after 3 days of treatment with levofloxacin (0.5 g, I.V., qd) combined with cefoperazone sodium sulbactam (3 g, I.V., q12h). A bronchoscopy was performed to obtain BALF for tNGS examination, which identified Cps. The patient’s medical history showed no obvious history of contact with birds or poultry. Levofloxacin and cefoperazone sodium sulbactam were discontinued and replaced with omadacycline (100 mg, I.V., qd). After 2 days of treatment, the patient’s fever subsided and his symptoms gradually improved. After 10 days of treatment, the patient was treated with doxycycline (100 mg, P.O., q12h). A chest CT scan showed that the pneumonia had been significantly reduced as compared to before.

### 3.6 Patient-6

A 70-year-old male was admitted to the hospital for 10 days of fever. He had fever every day with a maximum temperature of 38.9 °C, accompanied by diarrhea, muscle ache, and gradually developed cough. Initially, the symptoms improved after 5 days of ceftriaxone treatment. He had fever again 3 days ago, with a maximum temperature of 38.8 °C, accompanied by loss of appetite, so he came to our hospital for chest CT examination, which showed inflammation of the left upper lobe, local atelectasis, and a small amount of pleural effusion on both sides. Laboratory findings are summarized in Table 2. the fever persisted after 3 days of treatment with levofloxacin (0.5 g, I.V., qd). When he was admitted to the hospital, he underwent bronchoscopy to obtain BALF for tNGS examination, and the results showed Cps. After asking about the medical history, he kept chickens at home. Considering that the symptoms had improved, he continued the original treatment plan for 8 days and then changed to doxycycline (100 mg, P.O., q12h). The re-examination of chest CT showed that the pneumonia has significantly reduced as compared with the previous one.

### 3.7 Patient-7

A 63-year-old woman presented with fever and cough for 1 day, with a maximum temperature of 39.4 °C, accompanied by cough, decreased appetite, and myalgia. A chest CT at our hospital revealed pneumonia in the right lower lobe. Laboratory findings are summarized in Table 2. She was initially treated with levofloxacin (0.5 g, I.V., qd). However, after 3 days, the fever persisted. Bronchoscopy was performed, and the BALF was sent for tNGS, which identified Cps. Upon further questioning, a possible history of parrot exposure was identified. The treatment was then switched to doxycycline (100 mg, P.O., q12h). After 2 days, her fever subsided, and symptoms improved. A follow-up chest CT showed resolution of the pneumonia.

### 3.8 Patient-8

A 49-year-old woman presented with a 4 days history of cough and a 2 days history of fever (peaking at 39 °C), accompanied by fatigue, decreased appetite, and headache. After 2 days of outpatient treatment with ceftriaxone without improvement, she was admitted to our hospital. A chest CT revealed right lower lobe pneumonia and right-sided pleural effusion. Laboratory findings are summarized in Table 2. She was initially treated with levofloxacin (0.5 g, I.V., qd) combined with piperacillin-tazobactam (4.5 g, I.V., q8h). However, after 3 days, she remained febrile. Bronchoscopy was performed, and the BALF was sent for tNGS, which identified Cps. No clear history of bird or poultry exposure was identified upon further inquiry. Levofloxacin and piperacillin-tazobactam were discontinued, and therapy was switched to omadacycline (100 mg, I.V., qd). Her fever subsided, and symptoms improved after 2 days. After 8 days, treatment was transitioned to oral doxycycline (100 mg, P.O., q12h). The re-examination of chest CT showed that the pneumonia has significantly reduced as compared with the previous one.

### 3.9 Patient-9

A 60-year-old woman presented with fever and cough for 3 days, with a maximum temperature of 38.5 °C, accompanied by myalgia, blood-tinged sputum, and diarrhea. Initial outpatient treatment with moxifloxacin (400 mg, I.V., qd) for 3 days showed no improvement. Upon admission to our hospital, a chest CT revealed scattered bilateral pulmonary infiltrates. Laboratory findings are summarized in Table 2. She was started on cefoperazone-sulbactam (3 g, I.V., q12h), but after 3 days, her condition remained unchanged. Bronchoscopy was performed, and the BALF was sent for tNGS, which identified Cps. Further history-taking revealed possible poultry exposure. Cefoperazone-sulbactam was discontinued, and therapy was switched to omadacycline (100 mg, I.V., qd). Clinical improvement was noted within 3 days, with resolution of fever. After 7 days, treatment was transitioned to oral doxycycline (100 mg, P.O., q12h). The re-examination of chest CT showed that the pneumonia has significantly reduced as compared with the previous one.

### 3.10 Patient-10

A 66-year-old male presented with fever and cough for 6 days,with a maximum temperature of 38.7 °C, accompanied by decreased appetite and fatigue. Initial self-treatment with paracetamol/chlorphenamine/pseudoephedrine (phenylephrine) tablets proved ineffective. At our hospital, chest CT revealed right lower lobe pneumonia. Laboratory findings are summarized in Table 2. Empirical therapy with levofloxacin (0.5 g, I.V., qd) combined with cefoperazone-sulbactam (3 g, I.V., q12h) was initiated, but intermittent fever persisted after 5 days. Bronchoscopy was performed, and the BALF was sent for tNGS, which identified Cps. Upon detailed history-taking, parrot exposure was confirmed. Antimicrobial therapy was adjusted to omadacycline (100 mg, I.V., qd). Resolution of fever within 3 days and progressive symptom improvement. After 7 days, treatment was transitioned to oral doxycycline (100 mg, P.O., q12h). The re-examination of chest CT showed that the pneumonia has significantly reduced as compared with the previous one.

### 3.11 Patient-11

A 59-year-old male presented with a 3 days history of fever, with a maximum temperature of 38.7 °C, accompanied by shortness of breath, decreased appetite, and myalgia. Initial self-treatment with cephalosporin antibiotics were ineffective. Chest CT at our hospital revealed left lower lobe pneumonia with localized consolidation. Laboratory findings are shown in Table 2. After 3 days of levofloxacin treatment (0.5 g, I.V., qd) without improvement, bronchoscopy was performed, and BALF was sent for tNGS, which detected Cps. Further history-taking revealed exposure to poultry. Levofloxacin was discontinued, and omadacycline (100 mg, I.V., qd) was initiated. The patient’s fever improved after 2 days of treatment. After 9 days, therapy was switched to oral doxycycline (100 mg, P.O., q12h). Follow-up chest CT showed significant resolution of the pneumonia.

### 3.12 Patient-12

A 74-year-old male presented with fever and cough for 1 week, with a maximum temperature of 39.5 °C, accompanied by shortness of breath, fatigue, and myalgia. Initial Self-treatment with paracetamol/pseudoephedrine/dextromethorphan was ineffective. Chest CT at our hospital revealed right lower lobe pneumonia. After 3 days of levofloxacin (0.5 g, I.V., qd) with no improvement, bronchoscopy was performed, and BALF was sent for tNGS, which identified Cps and Aspergillus fumigatus. Further history-taking revealed suspected exposure to parrots. Levofloxacin was discontinued, and treatment was switched to omadacycline (100 mg, I.V., qd) combined with voriconazole (200 mg, P.O., q12h). The patient’s fever improved and symptoms alleviated after 3 days. After 10 days, therapy was transitioned to oral doxycycline (100 mg, P.O., q12h) combined with voriconazole (200 mg, P.O., q12h). Follow-up chest CT showed significant resolution of the pneumonia.

## 4 Clinical characteristics statistics

We can find from (Tables 3, 4) that among the 12 cases, only 3 was healthy, and the rest suffered from one or more chronic diseases. Only 2 case had no history of contact with birds or poultry, and the other 10 cases had a history of contact, including 7 cases of parrots and 3 case of chickens. All 12 patients had high fever, 9 had cough, 9 had muscle aches, 7 han Fatigue, 7 had muscle aches,3 had shortness of breath, 3 had diarrhea, 3 had headaches, 1 had vomiting, 1 had hematuria and 1 had blood-stained sputum. All 12 cases had hypoxemia, and 3 of them had type I respiratory failure. Furthermore, we can find from this that the higher the reads of *Chlamydia psittaci* in tNGS, the more likely it is to cause more severe symptoms, and the greater the chance of developing severe pneumonia.

**Table 3:** Basic information and clinical manifestations of 12 patients with psittacosis pneumonia.

Patient number	Age (years)/Gender	Underlying disease	Contact History	First symptoms	Associated symptoms	Complication	Maximum Body temperature (°C)	Severe pneumonia	Normalized read count of *Chlamydia psittaci*	Estimated pathogen load of *Chlamydia psittaci* (copies/mL)
Patient-1	47/Female	Hypertension	Parrot	High fever	Fatigue, muscle aches, headaches, shortness of breath	Hypoxemia, liver damage	39.9	No	NR	NR
Patient-2	73/Male	Hypertension, diabetes	Parrot	High fever and cough	Fatigue, muscle aches, loss of appetite	Type I respiratory failure, liver damage	40.1	No	7,716	5.5 × 10^5^
Patient-3	66/Female	Rheumatoid arthritis, Hypertension	Parrot	High fever and cough	A small amount of white sticky sputum, muscle aches, headache, vomiting	Hypoxemia, liver damage	39.8	No	NR	NR
Patient-4	55/Male	None	Parrot	High fever	Fatigue, loss of appetite	Hypoxemia	39.2	No	NR	NR
Patient-5	77/Male	Hypertension, gout, splenectomy	None	High fever and cough	Fatigue, muscle aches, hematuria, diarrhea	Type I respiratory failure, liver damage	39.8	Yes	11,637	>1.0 × 10^6^
Patient-6	70/Male	Colon cancer with liver metastasis	Chicken	High fever	Cough, diarrhea, muscle aches, loss of appetite	Hypoxemia, liver damage	38.9	No	256	4.3 × 10^3^
Patient-7	63/Female	splenectomy	Parrot	High fever and cough	muscle aches, loss of appetite	Hypoxemia	39.4	No	472	<1.0 × 10^3^
Patient-8	49/Female	None	None	High fever and cough	Fatigue, loss of appetite, headache	Hypoxemia, liver damage	39	No	43,922	>1.0 × 10^6^
Patient-9	60/Female	Fatty liver	Chicken	High fever and cough	muscle aches, diarrhea, blood-stained sputum	Hypoxemia, liver damage	38.5	No	754	7.4 × 10^3^
Patient-10	66/Male	None	Parrot	High fever and cough	Fatigue, loss of appetite	Hypoxemia, liver damage	38.7	No	22,989	>1.0 × 10^6^
Patient-11	59/Male	Colon cancer	Chicken	High fever	shortness of breath, muscle aches, loss of appetite	Hypoxemia	39.5	No	1,672	1.1 × 10^4^
Patient-12	74/Male	Chronic gastritis	Parrot	High fever and cough	shortness of breath, muscle aches, Fatigue	Type I respiratory failure, liver damage	39.5	Yes	10,376	>1.0 × 10^6^

NR, not reported (patient declined to provide information).

**Table 4:** Demographic characteristics and clinical manifestations.

Patient characteristics	Total (*n* = 12), *n* (%)	Value
Demographics		
Age (years)		63.25 ± 9.65
Male	7 (58.3%)	
History of contact	10 (83.3%)	
Underlying disease	9 (75.0%)	
Clinical manifestations		
Fever > 38.5 °C	12 (100.0%)	39.36 ± 0.51
Cough	9 (75.0%)	
Myalgia	9 (75.0%)	
Weakness	7 (58.3%)	
Anorexia	7 (58.3%)	
Dyspnea	3 (25.0%)	
Diarrhea	3 (25.0%)	
Headache	3 (25.0%)	
Vomiting	1 (8.3%)	
Hematuria	1 (8.3%)	
Hemoptysis	1 (8.3%)	
Type I respiratory failure	3 (75.0%)	
Hypoxemia	12 (100.0%)	
Severe pneumonia	2 (16.7%)	

Laboratory tests (Tables 2, 5) showed that 10 cases of white blood cells (WBC) were within the normal range (3.5–9.5×10^9^/L), and 2 cases were elevated. 11 cases of Lymphocytes (L) were below the normal range (1.1–3.2×10^9^/L). and 1 case were normol. C-reactive protein (CRP) was higher than the normal range (0-8 mg/L) in 12 cases to varying degrees. Procalcitonin(PCT)was within the normal range (<0.5ug/L) in 8 cases, and elevated in 4 cases. Lactate dehydrogenase (LDH) and creatine kinase (CK) were in the normal range (0–248 U/L, 0–136 U/L) in 4 case, and elevated in 8 cases. Albumin (ALB) was in the normal range (35–55 g/L) in 3 cases, and decreased in 9 cases. Blood sodium (NA) was lower than the normal range (137-–147 mmol/L) in 12 cases. Alanine aminotransferase (ALT) was in the normal range (0–35 U/L) in 7 cases, and elevated in 5 cases. Aspartate aminotransferase (AST) was in the normal range (0–35 U/L) in 4 cases, and elevated in 8 cases.

**Table 5:** Laboratory findings and radiologic features.

Characteristics	Patients, *n* (%)	Value
Laboratory findings		
Elevated WBC (3.5–9.5 × 10^9^/L)	2 (16.7%)	8.3 ± 3.1
Decreased Lymphocytes (1.1 ~ 3.2×10^9^/L)	11 (91.7%)	0.7 (0.6–0.8).
Elevated CRP (0–8 mg/L)	12 (100.0%)	138.9 ± 80.5
Elevated PCT (0–0.05 ng/mL)	4 (33.3%)	0.39 (0.17–0.64)
Elevated LDH (0–248 U/L)	8 (66.7%)	315 ± 116
Elevated CK (0–136 U/L)	8 (66.7%)	426 (84–1896)
Decreased ALB (35–55 g/L)	9 (75.0%)	34 (32–36)
Decreased NA (137-147 mmol/L)	12 (100.0%)	131 ± 4
Elevated ALT (0–35 U/L)	5 (41.7%)	45 ± 29
Elevated AST (0–35 U/L)	8 (66.7%)	46 ± 19
Lesion location		
Lower lobe involvement	10 (83.3%)	
Upper lobe involvement	2 (16.7%)	
Subpleural distribution	11 (91.7%)	
Imaging features		
Consolidation	10 (83.3%)	
Ground-glass opacity or patchy shadow	7(58.3%)	
Atelectasis	3(25.0%)	
Nodular opacity	1 (8.3%)	
Air bronchogram	11 (91.7%)	
Associated findings		
Pleural effusion	6 (50.0%)	
Hilar or mediastinal lymphadenopathy	6 (50.0%)	

Chest high-resolution computed tomography (HRCT) manifestations (Table 6): 10 cases of lesions were located in the lower lung, 2 cases in the upper lung, and 11 cases of lesions were located under the pleura. The morphology of the lesions was consolidation in 10 cases, ground glass or patchy shadows in 7 cases, atelectasis in 3 cases, nodular shadows in 1 case, and air bronchograms in 11 cases, 6 cases had pleural effusion and 6 cases had hilar or lymphadenopathy.

**Table 6:** Chest HRCT manifestations of 12 patients with psittacosis pneumonia.

Patient number	Lesion Location	Lesion morphology	Mainly subpleural distribution	Pleural effusion	Hilar or lymphadenopathy
Patient-1	Right lower lung	Ground-glass opacity, consolidation, and air bronchograms	Yes	Yes	Yes
Patient-2	Both lower lung	Atelectasis, consolidation, and air bronchograms	Yes	No	No
Patient-3	Both lower lung	Nodular shadows, air bronchograms can be seen in some cases	No	No	No
Patient-4	Left lower lung	Ground-glass opacity, patchy opacity	Yes	No	No
Patient-5	Right upper lung	Solid shadows, thickening of interlobular septa, and air bronchograms	Yes	Yes	Yes
Patient-6	Left upper lung	Atelectasis, ground-glass opacity, consolidation, and air bronchograms	Yes	Yes	No
Patient-7	Right lower lung	Ground-glass opacity, consolidation, and air bronchograms	Yes	No	No
Patient-8	Right lower lung	Ground-glass opacity, consolidation, and air bronchograms	Yes	Yes	Yes
Patient-9	Both lower lung	consolidation, patchy opacity, and air bronchograms	Yes	No	Yes
Patient-10	Right lower lung	Atelectasis, consolidation, and air bronchograms	Yes	No	No
Patient-11	Left lower lung	consolidation, and air bronchograms	Yes	Yes	Yes
Patient-12	Both lower lung	Ground-glass opacity, consolidation, and air bronchograms	Yes	Yes	Yes

## 5 Discussion

Psittacosis (Cps) pneumonia is rare in community-acquired pneumonia, accounting for only about 1% (2). The hosts of Cps are mostly parrots, but can also be poultry such as chickens and ducks. The pathogens mostly enter the human body through the respiratory tract to cause disease. The main manifestations after infection are non-specific flu-like symptoms or pneumonia, but the clinical manifestations may range from asymptomatic to severe complications (such as myocarditis, encephalitis, and multiple organ failure) (4). Human infection is mostly caused by contact with infected birds or poultry, and direct human-to-human transmission is extremely rare (1).

After Cps enters the human body, the incubation period is generally 5–14 days, and the elderly can reach 45 days. The infectious period is mostly 7–8 days after the onset of symptoms (1). The main cause is pneumonia, and the clinical manifestations are often high fever, cough, fatigue, muscle ache, etc. (5, 6). After Cps is inhaled through the lungs, it first enters the blood, proliferates in the liver, spleen, and mononuclear macrophage system, and then spreads to the whole-body organs through the blood, affecting the lungs, spleen, liver, kidneys, and central nervous system. Therefore, the clinical symptoms are diverse, including pneumonia, hepatitis, nephritis, renal function damage, etc. (7), and may also be complicated by gastroenteritis, meningitis, endocarditis, rash, etc. (8).

Due to its wide range of non-specific clinical manifestations, diagnosis usually requires a comprehensive judgment based on clinical manifestations, contact history (some patients also have no clear contact history), and etiological testing. Cps-infected people usually have normal or slightly elevated white blood cells, decreased lymphocyte counts or ratios, elevated CRP, and decreased blood sodium levels. ALB may also be decreased, LDH and CK may be increased, transaminases may be increased, and hypoxemia may occur. In severe cases, respiratory failure may occur (9–11). However, the above methods still cannot effectively distinguish it from other pneumonias (viruses, fungi, Legionella, etc.), so more specific detection methods are required for diagnosis. Real-time polymerase chain reaction (PCR) has replaced culture and is considered the gold standard for diagnosing *Chlamydia psittaci* (12). Therefore, it is of great significance to send sputum or BALF obtained under bronchoscopy for PCR testing in patients suspected of Cps pneumonia. Compared to metagenomics next generation sequencing (mNGS), tNGS uses a screening process, with specific library preparation and microbial sequence library (13). Targeted amplification can improve the reliability of pathogen detection, and only requires sequencing of specific target areas. Data processing and analysis are easier than mNGS. Therefore, tNGS has significant advantages in detection speed, sensitivity and specificity, and its cost is lower than mNGS, with good application potential (14).

In terms of drug treatment, tetracyclines, new tetracycline derivatives, macrolides and fluoroquinolones are currently the main drugs used to treat psittacosis. Since Cps does not have a typical cell wall, commonly used β-lactam antibiotics such as penicillin and cephalosporin are ineffective against it (15, 16). Tetracyclines are the first-line treatment for Cps infection, including doxycycline and minocycline. New tetracycline derivatives such as omadacycline and tigecycline have shown strong *in vitro* antibacterial activity against Cps. Omadacycline has high lung tissue concentration and good safety, and has become a new choice for the treatment of psittacosis. For pregnant women and those who have contraindication with tetracyclines, macrolide antibiotics including azithromycin and erythromycin, have good in vitro antibacterial activity against Cps and can be used as second-line treatment for psittacosis. Fluoroquinolones such as levofloxacin and moxifloxacin are second to tetracyclines and macrolides in efficacy. There are cases of treatment failure in clinical practice (17), so they are not recommended as the first choice of treatment. For critically ill patients, a combination therapy can be selected, with tetracyclines combined with macrolides or fluoroquinolones. At the same time, early use of glucocorticoids may have certain benefits for the prognosis of critically ill patients (18). If severe complications occur in patients with Cps infection, the complications should be dealt promptly and symptomatic supportive treatment should be provided while actively treating the primary disease.

## Data Availability

The original contributions presented in the study are included in the article/supplementary material, further inquiries can be directed to the corresponding author.
